# The Role of Continuous Theta Burst Stimulation on Primary Motor Cortex in Improving Bladder Function in Post-stroke Patients: A Case Report

**DOI:** 10.7759/cureus.56993

**Published:** 2024-03-26

**Authors:** Yao Qian, Yu Yao, Guilan Huang, Bin Su, Hewei Wang

**Affiliations:** 1 Department of Rehabilitation Medicine, Wuxi Central Rehabilitation Hospital, The Affiliated Mental Health Center of Jiangnan University, Wuxi, CHN; 2 Department of Rehabilitation Medicine, Huashan Hospital, Fudan University, Shanghai, CHN

**Keywords:** case report, noninvasive neuromodulation, primary motor cortex, continuous theta burst stimulation, neurogenic bladder, stroke

## Abstract

Neurogenic bladder (NB) is a frequently encountered post-stroke complication, characterized by symptoms, such as urinary incontinence, dysuria, increased frequency, and urgency. Here, we present a case of a 75-year-old male with urgent urination, frequent urination, urinary incontinence, conspicuous discomfort during urination, and an unpleasant smell in the urine following a stroke. By reviewing the patient's previous medical records of stroke and ruling out other potential causes for bladder dysfunction, a diagnosis of NB could be established. We implemented conventional physical therapy, pelvic floor muscle training with the electromyography biofeedback device, and continuous theta burst stimulation (cTBS) on the contralesional primary motor cortex area to manage bladder function. To the best of our knowledge, this is the first case report on cTBS applied to manage NB after stroke. Our treatment has demonstrated remarkable efficacy in enhancing bladder and kidney function, improving the overall quality of life, and alleviating anxiety and depression symptoms in this patient. This case study concludes that the noninvasive neuromodulation approach exhibits significant potential in the clinical field when addressing this specific patient population.

## Introduction

Neurogenic bladder (NB) is a commonly encountered complication following a stroke [1]. This condition brings about troublesome symptoms and complications for patients, such as urinary incontinence, dysuria, frequency, and urgency. Furthermore, NB exposes patients to an increased risk of developing upper urinary tract infections, urinary tract stones, renal fluid accumulation, and renal failure [2]. These complications significantly impact the psychological and emotional well-being of individuals affected [3]. In addition, NB has been identified as a significant prognostic indicator for mortality and unfavorable outcomes in stroke patients. Recent studies have demonstrated that the presence of urinary incontinence 30 days post-stroke can lead to a fourfold increase in the risk of one-year mortality among stroke survivors who maintain continence [4].

Currently, the main treatment methods for NB include intermittent catheterization, anticholinergic medication, pelvic floor muscle exercises, biofeedback techniques, transcutaneous sacral nerve stimulation, and posterior tibialis electrical stimulation [5,6]. Magnetic stimulation, commonly used for neuromodulation in the management of NB following spinal cord injury and Parkinson's disease, has not demonstrated clear efficacy in stroke [7,8]. In this case report, we present a patient who experienced urinary incontinence persisting for a duration of seven months subsequent to the stroke. We applied continuous theta burst stimulation (cTBS) to the primary motor cortex (M1) region, in conjunction with conventional interventions for bladder management. Remarkably, the patient exhibited highly favorable rehabilitation outcomes. This case report adheres to the CARE (CAse REports) guidelines.

## Case presentation

Patient information

A 75-year-old male, retired from his career as a middle school English teacher, was admitted to the medical facility due to a respiratory infection characterized by persistent coughing accompanied by sputum. In addition, he exhibited symptoms indicative of asthma and left limb dysfunction from an ischemic stroke about seven months ago. The patient experienced urinary and fecal incontinence within one week after the stroke, with a gradual return to normal defecation approximately three weeks later. Despite experiencing the desire to urinate, the patient encountered challenges in holding it. The patient was troubled by indications of the disorder affecting the lower urinary system, which included urgent urination, frequent urination, urinary incontinence, conspicuous discomfort during urination, and an unpleasant smell in the urine until he was re-admitted to the medical facility. The management of urinary output involved the utilization of receptacles for urine and the usage of absorbent garments, thereby further restricting the patient's autonomy. The doctor prescribed solifenacin succinate to him, but he refused to take it after one week due to experiencing dry mouth. The patient had a documented history of hypertension for over five years and a confirmed history of coronary heart disease for more than five years. There was no evidence of any familial or hereditary diseases. He was the father of two daughters and had a spouse who was in good health.

Clinical findings

During the physical examination, the long-term effects of a cerebral infarction that occurred seven months ago were observed. The Brunnstrom recovery stage assessment revealed that the patient's left upper limb, left hand, and left lower limb were categorized as stage IV, stage V, and stage IV, respectively. In addition, although the passive range of motion of the left limb was within normal limits, most of the muscle strength demonstrated a 3-grade rating by manual muscle test (Table 1). The patient was capable of temporarily standing for five minutes with light assistance from one therapist using a walker. The patient maintained normal cognitive function; however, in daily activities, the patient heavily relied on a wheelchair for mobility.

**Table 1:** Manual muscle testing outcomes

Joint	Muscle	Right (unaffected limb)	Left (affected limb)
Upper limb	Shoulder flexion	4/5	3-/5
	Shoulder extension	4/5	3-/5
	Shoulder abduction	4/5	3/5
	Shoulder adduction	4/5	3/5
	Elbow flexion	4/5	4-/5
	Elbow extension	4/5	4-/5
	Pronation	4/5	3/5
	Supination	4/5	3/5
	Wrist flexion	4/5	3/5
	Wrist extension	4/5	3/5
Lower limb	Hip flexion	4/5	3-/5
	Hip extension	4/5	2/5
	Hip abduction	4/5	3/5
	Hip adduction	4/5	3/5
	Knee flexion	4/5	3/5
	Knee extension	4/5	3/5
	Ankle plantarflexion	4/5	2+/5
	Ankle dorsiflexion	4/5	2+/5

The urodynamic investigation is necessary for detecting and specifying lower urinary tract dysfunction, with a grade A recommendation. We performed urodynamic testing on the patient to evaluate their urinary tract symptoms. In addition, we utilized the Neurogenic Bladder Symptom Scale to document the patient's symptoms and employed the Hospital Anxiety and Depression Scale to assess emotional problems. Lastly, we used the International Consultation on Incontinence Quality of Life Questionnaire Short Form to further appraise the patient's quality of life. Table 2 shows the results of the patient's comprehensive rehabilitation assessments.

**Table 2:** Rehabilitation assessment outcomes VAS, visual analog scale

The Brunnstrom recovery stages	IV- V- IV
Berg balance scale	27/56
Barthel index	37/100
Postvoid residual volume (ml)	180
Maximum detrusor pressure (mmH_2_O)	60
Maximum cystometric capacity (ml)	350
Bladder compliance (ml/mmH_2_O)	5.83
Neurogenic Bladder Symptom Scale	40
Pain VAS score during urination	7
International Consultation on Incontinence Quality of Life Questionnaire Short Form	7
Hospital Anxiety and Depression Scale	8

Timeline

Table 3 shows the description of the timeline of events.

**Table 3:** Timeline of events

Timeline	Events
March 23, 2023	The patient presented with left-sided limb weakness and was diagnosed with stroke.
October 20, 2023	The patient was admitted with symptoms of a lung infection and received drug interventions and conventional physical therapy.
October 30, 2023	The patient underwent a comprehensive assessment including urodynamic testing, neurogenic bladder symptom scale, Hospital Anxiety and Depression Scale, and Incontinence Quality of Life Questionnaire Short Form.
October 31, 2023	Neurogenic bladder was diagnosed.
November 1, 2023	Bladder function training, pelvic floor muscle training, and continuous theta burst stimulation started.
November 29, 2023	Physiotherapy was completed and follow-up was done.

Diagnostic assessment

**Figure 1:**
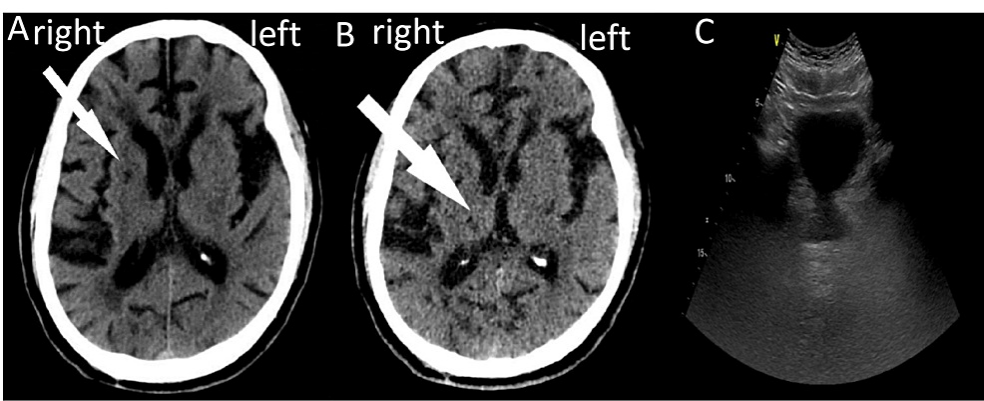
Computed tomography scan and prostate ultrasound A: right basal lacunar infarction, B: right thalamus lacunar infarction, C: normal prostatic ultrasound findings

On March 23, 2023, a computed tomography scan revealed the presence of ischemic stroke in the right basal ganglia and thalamus. This observation was subsequently determined as a diagnosis of stroke [9]. On October 30, 2023, an ultrasound examination was conducted to assess the morphology of the urinary system, revealing the kidneys, ureters, and prostate to exhibit normal structural characteristics (Figure 1). Furthermore, the results of a urine routine analysis exhibited an elevation in both the white blood cell count and bacteria count, surpassing the expected values within the normal range. In addition, urodynamic testing indicated increased detrusor pressure and overactive bladder. These findings, coupled with the frequent urination and other clinical symptoms, are suggestive of NB [1]. The presence of a sensation of burning during urination and abnormal results of urine routine indicate the presence of a urinary tract infection. The patient did not have any medical history, symptoms, or imaging findings that suggested the occurrence of spinal cord injury, spinal hernia, multiple sclerosis, or any other neurological disorder capable of causing NB.

Therapeutic interventions

After administering budesonide, compound ipratropium bromide atomized inhalation, and aminophylline oral as relevant medications to relieve symptoms of pulmonary infection, as well as incorporating conventional physical therapy and interventions targeting bladder function, we observed enhancements in both motor dysfunction and lower urinary tract symptoms. Specifically, these improvements were achieved through the utilization of bladder function training, pelvic floor muscle training with the electromyography biofeedback device, and the innovative application of cTBS on the contralesional upper limb M1 area (Table 4).

**Table 4:** Therapeutic interventions

Intervention		Dosage
Conventional physical therapy		Strength exercises, balance training, occupational therapy, 45 minutes/day
Bladder function training	Water consumption control training	Drink water regularly and consistently, about 400ml each time, 2000 ml in 24 hours
	Urination times	The interval between urination and water consumption was about two hours.
	Intermittent catheterization	Once every eight hours
Pelvic floor muscle training with the electromyography biofeedback device		10-second holds, 20 minutes per session, once daily for four weeks
Continuous theta burst stimulation on the contralesional primary motor cortex area		80% resting motor threshold, three pulses at 50 Hz repeated at 5 Hz, total pulse number 600, once daily for four weeks

The patient was positioned laterally following defecation for pelvic floor muscle training with biofeedback. An anal probe was inserted approximately 8 cm into the rectum to monitor the electrical activity of the anal sphincter. Two electrodes were placed 1 cm near the navel to measure the electrical activity of the abdominal muscles. The electromyogram (EMG) magnitude increased as the patient attempted to contract the sphincter. Once the electrical signal level reached a predetermined threshold, it would be displayed on the screen as an image signal, and videos would continue playing smoothly. Otherwise, if the threshold was not reached, video playback would be interrupted.

**Figure 2:**
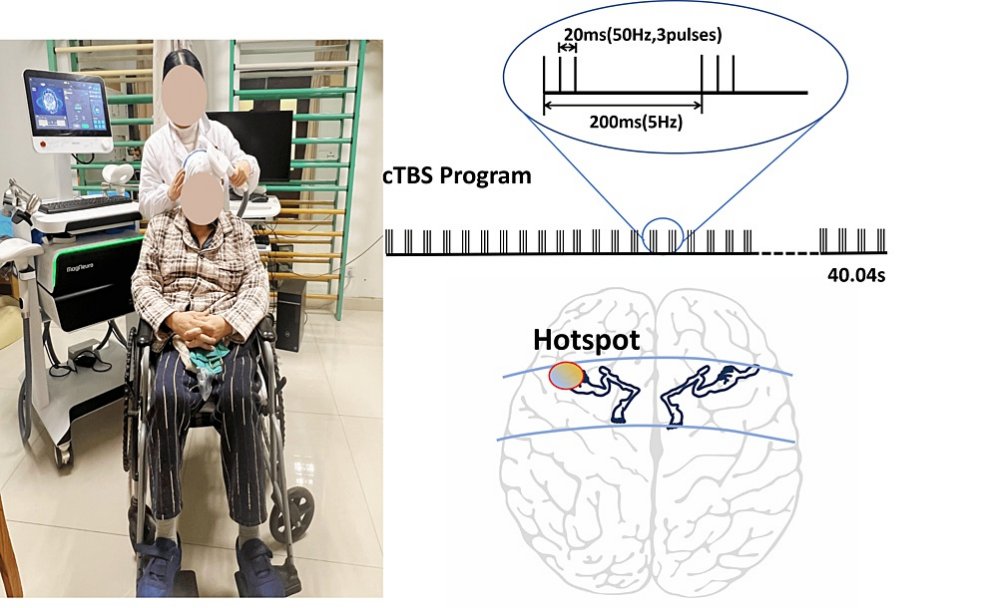
Continuous theta burst stimulation on contralesional primary motor cortex area cTBS, continuous theta burst stimulation

The Magneuro® (Vishee Medical Technology Co., Ltd., Nanjing, China) with a 70-mm figure-8-shaped stimulation coil was used to perform the cTBS protocol. The handle of the stimulation coil was placed at an angle of 45° posterior to the midline and placed over the contralesional M1. By measuring the active motion threshold of the contralesional M1, the stimulation intensity was determined at 80%. The cTBS paradigm consisting of three pulses at 50 Hz repeated at 5 Hz frequency was used, generating a total of 600 pulses in 40 seconds (Figure 2). The treatment was administered once daily, five times a week, for a duration of four weeks. Maintaining the precision of the stimulation site required patients to minimize cephalic movements. Therapists closely monitored for adverse reactions during treatment and took timely measures. All interventions were conducted with the informed consent of the patient.

Follow-up and outcome of interventions

After a four-week period, the identical set of assessments was conducted once more, and the results are presented in Table 5.

**Table 5:** Outcome measures “↑”, higher than normal range; VAS, visual analog scale

	Pretreatment	Posttreatment
Postvoid residual volume (ml)	180↑	50
Maximum detrusor pressure (mmH_2_O)	60↑	8
Maximum cystometric capacity (ml)	350	370
Bladder compliance (ml/mmH_2_O)	5.83	46.25
Urine creatinine（mmol/L）	8.8↑	4.4
White blood cell count (/μL)	41↑	3
Bacteria count (/μL)	561↑	225↑
Blood urea nitrogen (mmol/L)	8.38↑	6.54
Neurogenic Bladder Symptom Scale	40	24
Pain VAS score during urination	7	2
International Consultation on Incontinence Quality of Life Questionnaire Short Form	7	3
Hospital Anxiety and Depression Scale	8	1

After 20 sessions of cTBS, the urodynamic investigation revealed reduced detrusor contractility and normal bladder compliance in this patient. The urinalysis indicated normal values for urinary creatinine, and renal function has also returned to a normal level.

In addition, there was a significant decrease in symptom severity, as evidenced by notable reductions in residual urine volume, and white blood cell count. When the patient recently urinated, the pain decreased to a score of 2 and the unpleasant odor of urine vanished.

Moreover, the frequency of urinary incontinence decreased, leading to a significant reduction in the Neurogenic Bladder Symptom Scale. We also observed an improvement in the quality of life and a decrease in levels of anxiety and depression. Because the noninvasive nature of the interventions and the tolerable dosage of cTBS, this patient demonstrated excellent adherence with no reported adverse events during the treatment session.

## Discussion

As a specialized form of low-frequency repetitive transcranial magnetic stimulation, cTBS has the capacity to induce long-term depression-like plasticity, leading to a temporary suppression of cortical excitability in the targeted region for a duration of up to 60 minutes. Furthermore, cTBS not only reduces the excitability of the corticospinal tract but also inhibits intracortical facilitation [10]. In comparison to standard transcranial magnetic stimulation, the primary advantage of TBS mode lies in its multi-branched stimulation that accurately replicates the physiological state of neural activity. The therapeutic dosage only requires 80% of the motor threshold, thereby reducing the duration of stimulation to one-tenth of its original length. In addition, this type of stimulation demonstrates significant effectiveness in regulating nerves with minimal adverse effects. Therefore, cTBS efficiently addresses the clinical requirements of a substantial patient population undergoing daily treatment, greatly diminishing the duration required for transcranial magnetic stimulation while concurrently augmenting efficacy [11].

Micturition and storage are very complex processes. The brain, spinal cord, and peripheral nerves constitute a complex hierarchical neural network. The urination reflex is mediated by the spinal-bulbospinal-spinal reflex pathway, which is regulated by the pontine micturition center (PMC). During the urine storage phase, afferent signals from the bladder are transmitted to the periaqueductal gray via the spinal cord. Subsequently, inhibitory output from the periaqueductal gray is conveyed to the pontine region. When the afferent signal reaches a specific threshold, it triggers the reflex, leading to the excitation of PMC neurons. PMC neurons activate the parasympathetic nerves in the spinal cord, causing contraction of the detrusor muscle in the bladder and relaxation of the urethral sphincter. This bladder contraction elevates internal pressure and facilitates urine discharge through the urethra [12-14].

**Figure 3:**
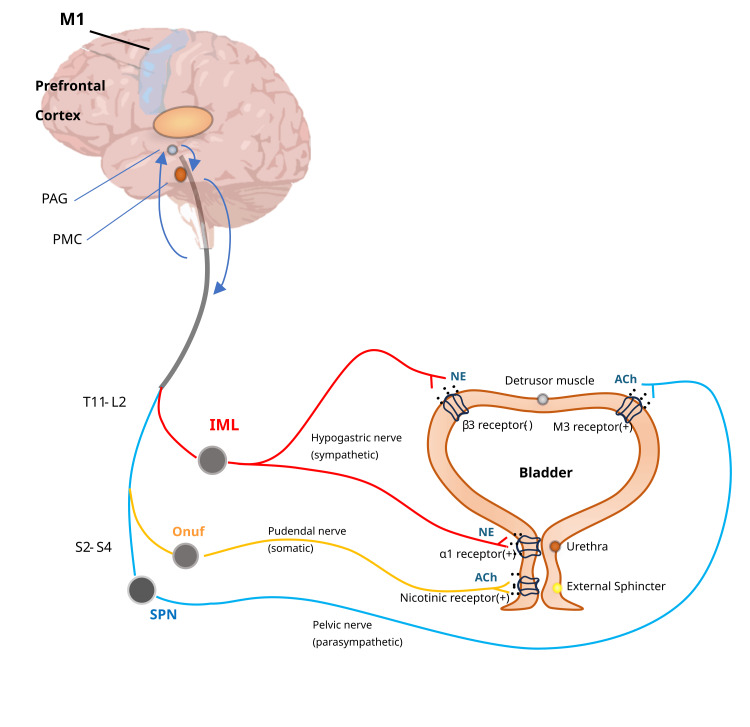
Neural control process of urination This is an original illustration. The primary motor cortex region possesses the capability to remotely regulate the PMC. The coordination of three neural systems controls the lower urinary tract, which includes the urinary bladder and its outlet. The sympathetic fibers (shown in red) originate in the T11-L2 intermediolateral cell column segments within the spinal cord. These fibers travel through the inferior mesenteric plexus and the hypogastric nerve or through the paravertebral chain to reach the pelvic nerves located at the base of the bladder and the urethra. The detrusor is controlled by the sympathetic system through the β3 receptor, while the bladder neck is regulated via the α1 receptor. Somatic motor nerves (shown in yellow) innervate the striated muscles of the external urethral sphincter and arise from S2-S4 Onuf's neurons. These nerves pass through the pudendal nerves. Parasympathetic preganglionic fibers (shown in blue) emerge from the S2-S4 sacral parasympathetic nucleus and travel through sacral roots and pelvic nerves to ganglia within the pelvic plexus and the bladder wall. Activation of the parasympathetic motor system leads to detrusor contraction through the M3 receptors. M1, primary motor cortex; PAG, periaqueductal gray; PMC: pontine micturition center; T, thoracic; L, lumbar; IML, intermediolateral cell column; Onuf, Onuf's nucleus; SPN, sacral parasympathetic nucleus; S, sacral; NE, norepinephrine; Ach, acetylcholine;α1, alpha-1 adrenergic receptor; β3, beta-3 adrenergic receptor

However, the precise location and function of cortical neurons controlling micturition from the higher regions of the brain have yet to be fully understood. The pseudorabies virus, which is retrogradely labeled, can be injected into the bladder, urethra, or external urethral sphincter of rats to label neurons in multiple brain regions upstream of PMC. These brain regions include the nucleus raphe, locus coeruleus, brain stem A5 cell group, medial preoptic area, paraventricular nucleus, lateral hypothalamic area, rubra, gigantocellular reticular nucleus, and some cerebral cortex regions. These brain regions are closely associated with the regulation of the lower urinary tract. Some researchers have discovered in mice that the M1 region has the ability to remotely regulate PMC. These neurons, predominantly located in the fifth layer, are identified as vertebral neurons based on their morphology (Figure 3) [15].

The impairment of any urinary pathway can disrupt urinary function, while stroke has the potential to affect various regions of brain tissues, leading to the complex pathogenesis of NB. Research findings indicate that applying low-frequency (1 Hz) repetitive transcranial magnetic stimulation on the primary motor cortex seems to exert inhibitory effects on bladder activity in individuals diagnosed with Parkinson's disease [8]. In our approach, we apply cTBS to the contralesional M1 in order to alleviate the symptoms of NB. Based on the model of interhemispheric competition inhibition, it is hypothesized that the affected motor cortex experiences inhibition due to increased transcallosal inhibition from the unaffected motor cortex [16]. Building upon this theoretical framework, we employed cTBS to specifically target the motor cortex of the unaffected upper limb in order to mitigate the inhibitory effect of the ipsilesional hemisphere.

To the best of our knowledge, this is the first case report on the application of continuous theta burst stimulation for managing NB after stroke. It is pleasing to observe the notable success of our intervention in augmenting bladder function, kidney function, and overall quality of life, as well as mitigating symptoms of anxiety and depression among the patients. According to Dionísio et al., when cTBS is applied over the contralesional primary motor cortex of subacute post-stroke patients (within 7 ± 3 days), it results in an increase in cortical excitability and motor function in the ipsilesional hemisphere [17]. As a result, the patient's muscle strength and balance showed improvement, although it is important to note that this outcome can be attributed to a combination of conventional physical therapy and cTBS.

The current body of literature indicates that pelvic floor muscle training has been shown to alleviate symptoms, such as frequent urination, urgency to urinate, and incontinence in patients [18]. However, there is a lack of evidence supporting the reduction of detrusor pressure. According to Lúcio et al., the group that underwent pelvic floor muscle training and transcutaneous tibial nerve stimulation experienced a significant decrease in detrusor pressure at maximum flow rate after 12 weeks of treatment in comparison to their initial measurements. Conversely, the group that received pelvic floor muscle training and sham neuromuscular electrical stimulation did not exhibit a significant change in this particular index [19]. The preservation of upper urinary tract function is the main focus throughout the diagnosis, evaluation, and follow-up of neurogenic lower urinary tract dysfunction (clinical principle) [20]. Based on the principles of cTBS, it is reasonable to infer that the intervention reduced detrusor pressure, prevents urine reflux, and safeguards the function of the upper urinary tract.

However, it is important to acknowledge certain limitations in this case study. For instance, while cTBS was employed on the upper limb M1 area during treatment, further investigation is still required to determine the impact of different magnetic stimulation on other cortical regions. In addition, further clinical randomized controlled trials are imperative to validate the efficacy of cTBS.

## Conclusions

This case report presents a new rehabilitation method for managing NB after stroke through the use of noninvasive neuromodulation. The patient expressed contentment with the therapeutic procedure and the favorable outcomes in this report. Continuous theta burst stimulation on the primary motor cortex combined with bladder function training and pelvic floor muscle training may help to improve bladder function after stroke. This noninvasive neuromodulation approach exhibits significant potential in the clinical field when addressing this specific patient population. However, further high-quality clinical studies are required to confirm its role in NB after stroke.
